# Phase plane dynamics of ERK phosphorylation

**DOI:** 10.1016/j.jbc.2023.105234

**Published:** 2023-09-09

**Authors:** Stanislav Y. Shvartsman, Sarah McFann, Martin Wühr, Boris Y. Rubinstein

**Affiliations:** 1 Department of Molecular Biology, Princeton University, Princeton, New Jersey, USA; 2 Lewis-Sigler Institute for Integrative Genomics, Princeton University, Princeton, New Jersey, USA; 3 Center for Computational Biology, Flatiron Institute, New York, New York, USA; 4 Department of Chemical and Biological Engineering, Princeton University, Princeton, New Jersey, USA; 5 Stowers Institute for Medical Research, Kansas City, Missouri, USA

**Keywords:** mitogen-activated protein kinase (MAPK) signaling, enzyme kinetics, modeling and simulation, dual phosphorylation, parameter estimation, phase plane analysis, heterozygosity

## Abstract

The extracellular signal–regulated kinase (ERK) controls multiple critical processes in the cell and is deregulated in human cancers, congenital abnormalities, immune diseases, and neurodevelopmental syndromes. Catalytic activity of ERK requires dual phosphorylation by an upstream kinase, in a mechanism that can be described by two sequential Michaelis-Menten steps. The estimation of individual reaction rate constants from kinetic data in the full mechanism has proved challenging. Here, we present an analytically tractable approach to parameter estimation that is based on the phase plane representation of ERK activation and yields two combinations of six reaction rate constants in the detailed mechanism. These combinations correspond to the ratio of the specificities of two consecutive phosphorylations and the probability that monophosphorylated substrate does not dissociate from the enzyme before the second phosphorylation. The presented approach offers a language for comparing the effects of mutations that disrupt ERK activation and function *in vivo*. As an illustration, we use phase plane representation to analyze dual phosphorylation under heterozygous conditions, when two enzyme variants compete for the same substrate.

Several essential processes in the cell, including protein synthesis, gene regulation, and energy metabolism, require activity of the extracellular signal–regulated kinase (ERK) (1, 2). This highly conserved enzyme is activated by cell surface receptors, such as receptor tyrosine kinases, which trigger activation of a dual specificity kinase, mitogen-activated ERK kinase (MEK), which has ERK as its only substrate (3, 4, 5). MEK phosphorylates, in strict order, tyrosine and threonine residues in the TEY sequence within the activation loop of ERK. These phosphorylations induce a conformational change which activates ERK and cause dissociation of the ERK/MEK complex, enabling ERK to phosphorylate a broad spectrum of substrates (Fig. 1*A*) (6). Given its critical roles in cell regulation, it is unsurprising that mutations affecting either MEK or ERK can lead to diseases. Indeed, dozens of MEK and ERK variants have been documented in human cancers, congenital abnormalities, immune diseases, and neurodevelopmental syndromes (7, 8, 9, 10, 11).

**Figure 1:**
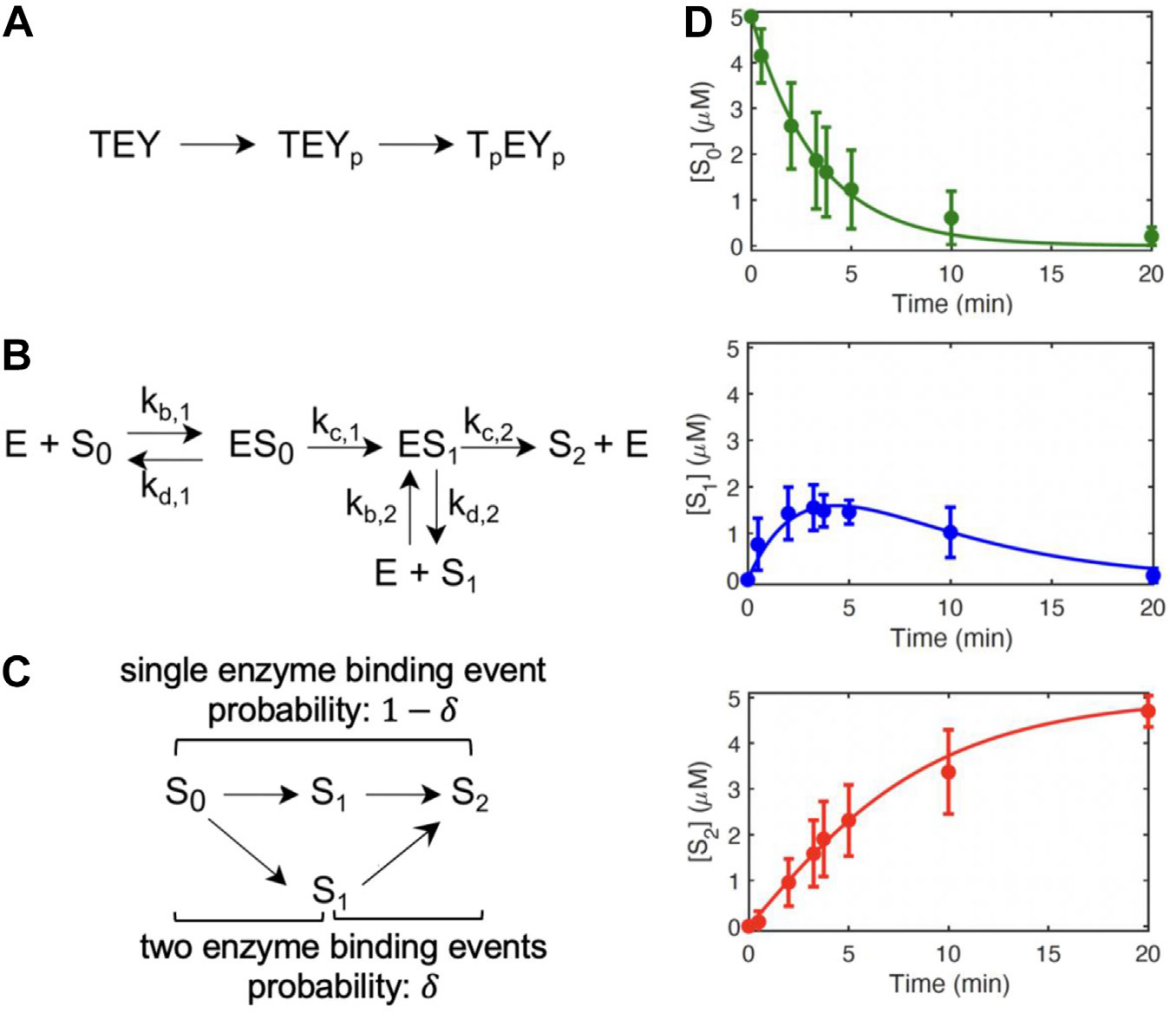
**Kinetic mechanism of dual phosphorylation of ERK by active MEK.***A*, MEK activates ERK by ordered dual phosphorylation of tyrosine and threonine amino acids within the activation loop of ERK. *B*, mechanism of ERK activation by MEK can be modeled by two sequential Michaelis-Menten steps. *C*, schematic representation of processive and distributive dual phosphorylation channels. *D*, time courses showing the concentrations of unphosphorylated (S_0_), monophosphorylated (S_1_), and dually phosphorylated ERK (S_2_) generated by a mixture of 0.67 μM of activated MEK, 0.67 μM total ERK, and 5 mM ATP. S_0_, S_1_, and S_2_ concentrations were monitored using phos-tag gel electrophoresis, as described in Yeung *et al.* (19). The error bars indicate the standard deviation of 12 replicates. Data are from Yeung *et al.* (19). ERK, extracellular signal–regulated kinase.

Most pathogenic variants of MEK and ERK have amino acid substitutions in different parts of these proteins. Investigating the effects of these sequence changes leads to questions at multiple levels of biological organization (7, 12, 13, 14, 15): What happens to the structure and dynamics of the molecule harboring a substitution? What are the effects of these changes on the mechanisms and rates of reactions in which this molecule participates? How do these changes affect the dynamics of biochemical networks constructed from these reactions? What are the effects of network-level changes on cellular processes, such as cell differentiation and growth? Going further, what are the effects of cellular level changes on tissues and organs?

The dynamics of ERK phosphorylation follow a simplified mechanism with two sequential Michaelis-Menten steps (Fig. 1, *B* and *C*) (4). The simplifications include a coarse-grained description of the ERK/MEK association and ATP/ADP exchange. Even with these simplifications, there are six rate constants that prove hard to estimate with high confidence based on the measured time courses (16). This difficulty in solving the inverse kinetics problem is a generic feature of multiparameter dynamic models (17). Here, we offer a parameter estimation approach that addresses some of these challenges, focusing on studies that reconstitute ERK regulation *in vitro* (18). We focus on experiments in which preactivated MEK, either wildtype or containing a pathogenic substitution, is added to a mixture of unphosphorylated ERK with excess ATP, initiating gradual conversion of ERK to the dually phosphorylated state (Fig. 1*D*) (19).

In contrast to most parameter estimation studies, which fit the time series data, we use the phase plane representation of dynamics. The term phase plane is from classical mechanics, where the state of a system with N particles is fully described by a point in 6N-dimensional phase space, where coordinates correspond to particles’ positions and velocities. For a single particle moving in one dimension, the phase space becomes a phase plane. To make things concrete, consider a ball that moves under the action of gravity after being thrown upwards from height $y_{0}$ with initial velocity $v_{0}$. The time-dependent trajectory of this ball traces out the following curve in the phase plane: ${y\left(v\right)=y}_{0}+{v_{0}}^{2}/2g-v^{2}/2g$, which is of course a locus of constant energy and can be derived without having explicit expression for the time-dependence of the ball’s velocity and position. Below, we derive the phase plane representation for the temporal progress of ERK phosphorylation by MEK and demonstrate how it can be used to analyze kinetic data and interpret the effects of mutations.

## Results

### Phase plane dynamics of dual phosphorylation

We start with a mass-action model of a kinetic experiment in which active MEK is added to unphosphorylated ERK (4, 5). The concentrations of MEK and ERK are denoted by $E_{T}$ and $S_{T}$, respectively. The composition of the reaction mixture is described by six concentrations, corresponding to the free enzyme (E), three phosphorylation states ($S_{0}$, $S_{1},S_{2}$), and two enzyme–substrate complexes ($C_{0}$, $C_{1}$). Since ERK activation follows an ordered mechanism, $S_{1}$ corresponds to ERK phosphorylated on a tyrosine residue within the activation loop (Fig. 1, *A* and *B*). When monophosphorylated substrate does not dissociate from the enzyme before the next phosphorylation, dual phosphorylation is called processive (4, 20); otherwise, it is called distributive (Fig. 1*C*).

At the start of the experiment, $S_{0}\left(0\right)$ = $S_{T}$, E(0) = $E_{T}$, and all other concentrations are equal to zero. Since there are two conservation laws: $E_{T}=E\left(t\right)+C_{0}\left(t\right)+C_{1}\left(t\right)$ and $S_{T}=S_{0}\left(t\right)+S_{1}\left(t\right)+S_{2}\left(t\right)+C_{0}\left(t\right)+C_{1}\left(t\right)$, the composition of the system is described by four differential equations:

$$\frac{dS_{0}}{dt}=-k_{b,1}S_{0}\times E+k_{d,1}C_{0}{,S}_{0}\left(0\right){=S}_{T,}$$

$$\frac{dC_{0}}{dt}=k_{b,1}S_{0}\times E-k_{d,1}C_{0}-k_{c,1}C_{0}{,C}_{0}\left(0\right)=0\text{,}$$

$$\frac{dC_{1}}{dt}=k_{b,2}S_{1}\times E-k_{d,2}C_{1}+k_{c,1}C_{0}-k_{c,2}C_{1}{,C}_{1}\left(0\right)=0\text{,}$$

$$\frac{dS_{1}}{dt}=-k_{b,2}S_{1}\times E+k_{d,2}C_{1}{,S}_{1}\left(0\right)=0\text{,}$$

with E(t) and $S_{2}\left(t\right)$ obtained from the conservation laws.

Next, we use a steady-state approximation for complexes, which is valid when $S_{T}\gg E_{T}$ (16, 21), and rescale the problem as follows: $\tau \equiv t\times k_{c,1}\times \frac{E_{T}}{S_{T}},x\equiv \frac{S_{0}}{S_{T}},y\equiv \frac{S_{1}}{S_{T}}$. This leads to the following reduced model:

$$\frac{dx}{d\tau }=-\frac{x}{\alpha x+\beta y+\gamma },\;x\left(0\right)=1\text{,}$$

$$\frac{dy}{d\tau }=\frac{\delta x-\varepsilon y}{\alpha x+\beta y+\gamma },\;y\left(0\right)=0\text{.}$$

The five dimensionless parameters in these equations are defined as follows:

$$\alpha \equiv \frac{k_{c,1}+k_{c,2}+k_{d,2}}{k_{c,2}+k_{d,2}},\beta \equiv \frac{K_{M,1}}{K_{M,2}},\gamma \equiv \frac{K_{M,1}}{S_{T}},\delta \equiv \frac{k_{d,2}}{k_{c,2}+k_{d,2}},\varepsilon \equiv \left(\frac{k_{c,2}}{K_{M,2}}\right)/\left(\frac{k_{c,1}}{K_{M,1}}\right)\text{,}$$

where $K_{M,1\left(2\right)}$ are the Michaelis constants of the two phosphorylation steps: $K_{M,1\left(2\right)}=\frac{k_{c,1\left(2\right)}+k_{d,1\left(2\right)}}{k_{b,1\left(2\right)}}$.

The two equations can be combined into a single ordinary differential equation for the dependence of the monophosphorylated concentration on the unphosphorylated concentration:

$$\frac{dy}{dx}=\varepsilon \frac{y}{x}-\delta ,\;y\left(1\right)=0\text{.}$$

This equation has a closed form solution:

$$y\left(x;\delta ,\varepsilon \right)=\frac{\delta }{1-\varepsilon }\left(x^{\varepsilon }-x\right)\text{,}$$

which describes the trajectory that joins the fully unphosphorylated and dually phosphorylated states, going through a peak at $x_{\max}=\varepsilon^{\frac{1}{1-\varepsilon }},y_{\max}={\delta \varepsilon }^{\frac{\varepsilon }{1-\varepsilon }}$.

The derived expression depends on two dimensionless groups. The first group, δ, is the probability that the newly formed complex of the enzyme and monophosphorylated substrate dissociates before the substrate is phosphorylated the second time (22, 23, 24). Note that 1−δ is the probability that both phosphorylations happen within the same enzyme–substrate binding event (also known as the processivity). As a consequence, δ is the probability of the distributive reaction channel. The second group, ε, is the ratio of the second-order rate constants (also known as enzyme specificities) for two of the enzymatic reactions, quantifying how the unphosphorylated and monophosphorylated substrates compete for their common enzyme. Fig. S1 shows the plots of the derived expression for different choices of δ and ε. We see that δ simply scales the curve the shape of which is determined by ε.

### Using phase plane representation for parameter estimation

**Figure 2:**
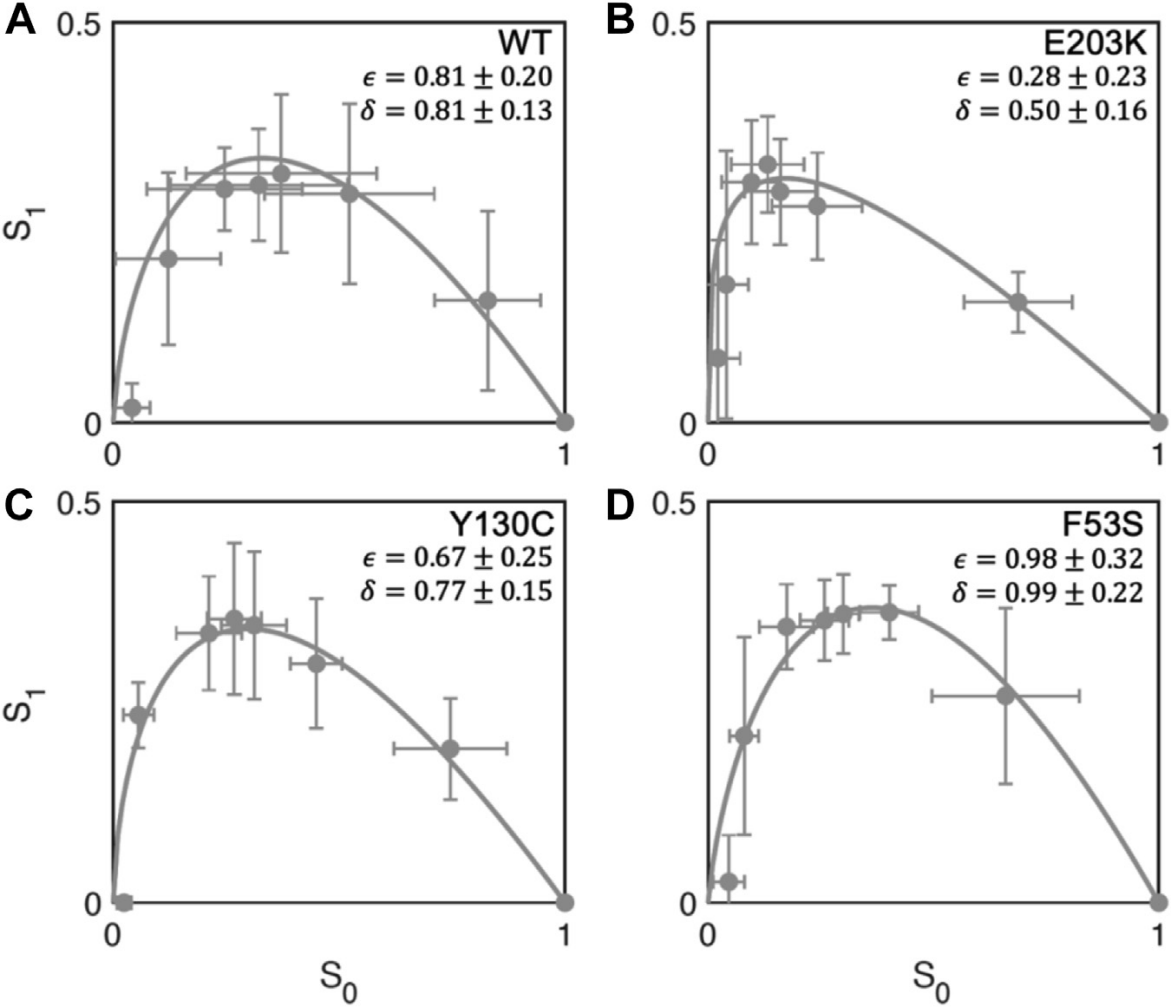
**Two-parameter model fit to ERK phosphorylation trajectories.***A*, phase plane trajectory of ERK phosphorylation by WT MEK (*gray*). Average S_0_ and S_1_ values are plotted for seven time points. The standard deviation in S_0_ and S_1_ for each time point is shown (N_WT_ = 12). *The solid line* represents the model fit to data. S_0_ and S_1_ represent unphosphorylated and monophosphorylated ERK, respectively, normalized by the total amount of substrate. *B*–*D*, model fits to the E203K, Y130C, and F53S trajectories (N_E203K_ = 5; N_Y130C_ = 5; N_F53S_ = 5). Best fit values are displayed along with their 95% confidence intervals. ERK, extracellular signal–regulated kinase.

The phase plane trajectory, which uses a single curve to show how the amounts of unphosphorylated and monophosphorylated ERK vary in relation to one another across time (Figs. 2, *A*–*D* and S2, *A* and *B*), provides a convenient way for analyzing the kinetics of ERK phosphorylation. The convenience comes from the fact that it is insensitive to errors in estimating the times at which the reaction has been stopped and is independent of γ, both of which depend on the experimental choice of substrate concentration. The probability of the distributive reaction channel (δ) and the ratio of the two specificities (ε) can be readily estimated from kinetic data. As an illustration, we show the nonlinear least squares fits to the data from our earlier experiments where ERK was phosphorylated by either wildtype MEK or one of the three MEK variants from human diseases (19, 25, 26). Each of these variants has a single amino acid substitution: the E203K variant was identified in melanoma (Fig. 2*B*), while the Y130C and F53S variants were identified in the cardiofaciocutaneous syndrome (Fig. 2, *C* and *D*). The cancer mutant has a strongly reduced probability of distributed phosphorylation, whereas the cardiofaciocutaneous syndrome variants are indistinguishable from the wildtype, at least with respect to their ability to directly phosphorylate ERK (Fig. 2, *A*–*D*). Understanding these effects *in vivo* requires carrying out similar analyses for other reactions involving MEK, including their activation by Raf, dephosphorylation by phosphatases, and degradation by proteases (7, 27, 28).

**Figure 3:**
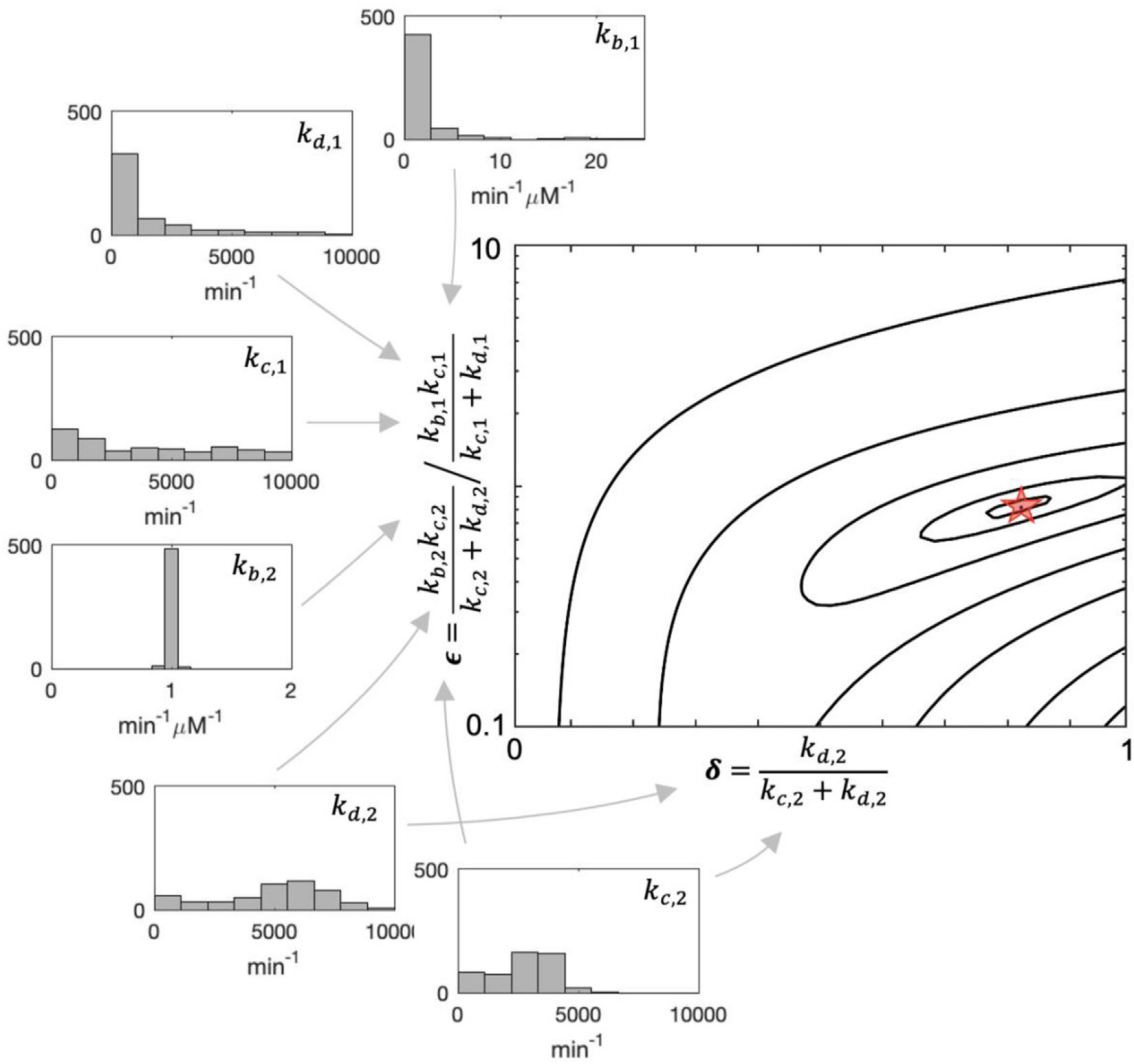
**Summary of nonlinear least square fits with the six-parameter and two-parameter models.** The six histograms labeled $k_{b,1}$, $k_{d,1}$, $k_{c,1}$, $k_{b,2}$, $k_{d,2}$, and $k_{c,2}$ show the marginal distributions of each parameter obtained by fitting the six-parameter model to wildtype MEK data. The central contour plot corresponds to the best fit using a 2-parameter model. *Gray arrows* indicate which parameters from the original model contribute to the two parameters of the reduced model. The *contour plot axes* represent the values of each of the two parameters, and *contour lines* correspond to different values of the squared norm of the residual for 96 data points (12 replicates, 7 time points per replicate) of the wildtype MEK data. The *red dot* at δ = 0.81, ϵ = 0.81 marks the best fit for wildtype MEK.

The proposed approach to kinetic data analysis will not work when a mutation changes the mechanism of phosphorylation from a strictly ordered to random, and the dynamics can no longer be analyzed in terms of a two-dimensional trajectory. However, it shows how one can make progress in model-based data analysis even when individual rate constants cannot be constrained. One can appreciate this advance by examining the results of nonlinear fits to data using the full, six-parameter model. To that end, we sampled six-dimensional parameter space to generate the initial guesses for the nonlinear least squares algorithm that converged to local minima of the objective function. Figure 3 displays the marginal distribution functions for the six rate constants from multiple minima identified by the algorithm. The individual rate constants are not constrained, which makes the full model not useful for comparing different variants, highlighting the value of the presented phase plane analysis.

The linearized form of the model is obtained when αx+βy≪γ. This case is realized when most enzyme molecules are not bound to substrates, and their concentration can be assumed constant throughout the transformation from the unphosphorylated to dually phosphorylated state. In this case, the equations for the three phosphostates become:

$$\frac{dS_{0}}{dt}=-\kappa_{1}S_{0}{,S}_{0}\left(0\right){=S}_{T}\text{,}$$

$$\frac{dS_{1}}{dt}=-\kappa_{2}S_{1}+{\delta \kappa }_{1}S_{0}{,S}_{1}\left(0\right)=0\text{,}$$

$$\frac{dS_{2}}{dt}=\kappa_{2}S_{1}+\left(1-\delta \right)\kappa_{1}S_{0}{,S}_{2}\left(0\right)=0\text{,}$$

where $\kappa_{1,2}\equiv \frac{k_{c,1\left(2\right)}}{K_{M,1\left(2\right)}}E_{T}$. The linear model can be used to fit the time series, yielding the estimates for the specificities of individual phosphorylation steps and the probability of the distributive reaction channel (19). The validity of this model depends on the assumption of linearity, which might be hard to justify. We realized that since our phase plane approach is free from these assumptions, it can provide an independent test of the linear model. Specifically, when the values of δ and ε obtained by fitting the data as a phase plane trajectory and as time series are close to each other, we have an additional argument in favor of the linear model. We found that this is indeed the case. Fitting the linear model to the wildtype time series data, we found that both mean and standard deviation values for δ (0.80, 0.18) and ε (0.80,0.53) are close to the wildtype values shown in Figure 2*A*. This justifies the use of a linear model in our earlier work (19).

### Phase plane analysis of heterozygous systems

**Figure 4:**
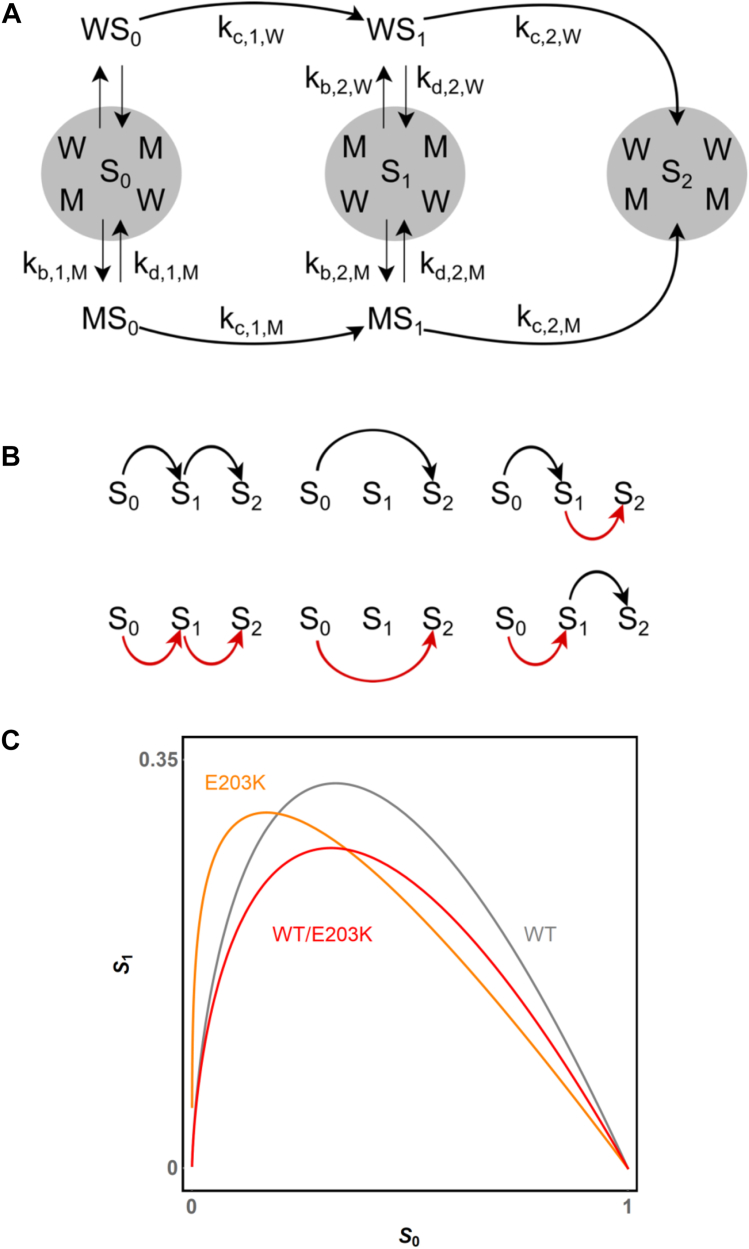
**Dual phosphorylation in heterozygous conditions.***A*, kinetic model of dual phosphorylation of a single substrate by an equimolar mixture of the wildtype (W) and mutant (M) enzyme variants. Each phosphorylation step is still modeled by a Michaelis-Menten mechanism but can be carried by either the wildtype of mutant enzyme variants. *B*, schematic representation of six paths connecting the unphosphorylated (S0) and dually phosphorylated (S2) phosphostates. *C*, predicted phase plane trajectory of the 1:1 mixture of the wildtype and E203K MEK variants (*red*) plotted together with the phase trajectories of pure wildtype (*black*) and pure E203K MEK (*orange*).

Once kinetic properties of individual variants are understood, one can ask what happens when two different variants are present in the same reaction system, as occurs in the heterozygous conditions in diseases associated with gain-of-function mutations in MEK (Fig. 4*A*) (29). The fact that even a single copy of a mutant gene can cause a phenotype poses interesting theoretical questions about heterozygous enzyme networks. Most modeling papers on heterozygosity focus on loss-of-function mutations (30, 31, 32, 33). Such systems can be adequately modeled by changing the enzyme (or substrate) dosage, which means that models established for the homozygous conditions still hold but operate in a different parameter regime. On the other hand, when two different variants are simultaneously present, the model is different as one must consider more species and reaction paths. This is true even for the simple case of ordered ERK phosphorylation. When only one variant is present, an ERK molecule can arrive to its dually phosphorylated state *via* two different paths, depending on whether it dissociates from MEK after the first phosphorylation. In a heterozygous mixture, both of these paths are present (for each variant), but there are also two new paths, in which the first and second phosphorylation steps are carried out by different enzyme variants (Fig. 4*B*).

Our phase plane analysis for homozygous systems can be applied to heterozygous conditions. Consider a mixture in which two MEK variants (wildtype, “w,” with total concentration $W_{T}$, and mutant “m,” with total concentration $M_{T}$) are phosphorylating ERK. Using the same nondimensionalization as for the wildtype system and eliminating the enzyme–substrate complexes, we arrive at the following model for the nondimensionalized concentrations of the unphosphorylated and monophosphorylated substrates:

$$\frac{dx}{d\tau }=-\left(\frac{x}{\alpha_{w}x+\beta_{w}y+\gamma_{w}}+\rho \frac{x}{\alpha_{m}x+\beta_{m}y+\gamma_{m}}\right),x\left(0\right)=1\text{,}$$

$$\frac{dy}{d\tau }=\left(\frac{\delta_{w}x-\varepsilon_{w}y}{\alpha_{w}x+\beta_{w}y+\gamma_{w}}+\rho \frac{\delta_{m}x-\varepsilon_{m}y}{\alpha_{m}x+\beta_{m}y+\gamma_{m}}\right),y\left(0\right)=0\text{.}$$

All subscripted parameters have the same meanings as before but must be evaluated for a specific variant. The new dimensionless group $\rho \equiv \frac{M_{T}}{W_{T}}\frac{k_{c,1,m}}{k_{c,1,w}}$ quantifies the relative amounts of two variants, and the relative rates constants for the first catalytic steps. The phase plane dynamics for this system obeys the following differential equation:

$$x\frac{dy}{dx}=-\frac{\left(\delta_{w}x-\varepsilon_{w}y\right)\left(\alpha_{m}x+\beta_{m}y+\gamma_{m}\right)+\rho \left(\delta_{m}x-\varepsilon_{m}y\right)\left(\alpha_{w}x+\beta_{w}y+\gamma_{w}\right)}{\rho \left(\alpha_{w}x+\beta_{w}y+\gamma_{w}\right)+\left(\alpha_{m}x+\beta_{m}y+\gamma_{m}\right)},y\left(1\right)=0\text{.}$$

While we could not find a closed form solution, we can show that, as before, the trajectory starts at (1,0) and ends at (0,0), going through a maximum ${(x}_{\max}^{het},y_{\max}^{het})$.

Can this trajectory be approximated by a trajectory of an appropriately chosen homozygous system? We start with the case when both enzyme variants are operating far from saturation, which corresponds to $\gamma_{w},\gamma_{m}\gg 1\text{.}$ The phase plane representation of the heterozygous dynamics can be shown to satisfy:

$$\frac{dy}{dx}=\varepsilon_{het}\frac{y}{x}-\delta_{het},y\left(1\right)=0\text{,}$$

where $\varepsilon_{het}=\frac{\varepsilon_{w}+\rho \varepsilon_{m}\left(\gamma_{w}/\gamma_{m}\right)}{1+\rho \left(\gamma_{w}/\gamma_{m}\right)}$ and $\delta_{het}=\frac{\delta_{w}+\rho \delta_{m}\left(\gamma_{w}/\gamma_{m}\right)}{1+\rho \left(\gamma_{w}/\gamma_{m}\right)}$. Thus, in this regime, there is an explicit connection between the effective parameters of individual variants and parameters of the effective homozygous model. Moreover, as ρ is varied from zero to infinity, the effective parameters are linearly transformed between parameters of individual variants.

To illustrate the derived expression for the effective heterozygous parameters, we predicted the phase plane trajectory of the heterozygous mixture of the wildtype and E203K variants, which display significant differences in processivity. To compute the heterozygous trajectory, we set ρ=1, since the protein levels are the same, and took the limit when both $\gamma_{m}$ and $\gamma_{w}$ are large, since the quality of the linear model was found acceptable. The peak of the heterozygous trajectory is close to the peak of the wildtype, but the amplitude is smaller.

In general case, the phase plane trajectory for the heterozygous system can be found numerically, along with the corresponding value for ${(x}_{\max}^{het},y_{\max}^{het})\text{.}$ One can then use this value to find the ($\varepsilon_{het},\delta_{het}$) pair that predicts the phase trajectory with the same maximum:

$$x_{\max}^{het}={\varepsilon_{het}}^{\frac{1}{1-\varepsilon_{het}}},y_{\max}^{het}=\delta_{het}{\varepsilon_{het}}^{\frac{\varepsilon_{het}}{1-\varepsilon_{het}}}\text{.}$$

Effectively, this procedure approximates dynamics of the 11-parameter heterozygous system by the 2-parameter homozygous system, although the explicit connection between ($\varepsilon_{het},\delta_{het}$) and parameters of the individual variants is lost. We evaluated the quality of this approximation by randomly generating heterozygous systems, finding the maxima of their phase plane trajectories, matching them by effective homozygous systems, and calculating the relative error; the quality of this approximation was excellent. We therefore conclude that heterozygous dynamics for this mechanism is always well approximated by an appropriately chosen homozygous model.

## Discussion

For one-step enzymatic reactions, an enzyme/substrate pair is characterized by its specificity constant, $k_{cat}/K_{M}$, which provides a quantitative way for comparison of enzyme/substrate pairs. In writing this paper, we were motivated to understand what metrics can be used for such comparisons in more complex mechanisms, starting with a realistic case of two sequential phosphorylations by the same kinase. Our phase plane representation of dual phosphorylation dynamics identified two parameters that can be used to compare enzyme/substrate pairs, as long as they follow the same mechanism. One of them is the ratio of two enzyme specificities, the other is the probability of the distributive reaction channel. We illustrated our approach by analyzing data of ERK phosphorylation by the wildtype and variant versions of MEK and suggest that it is already suitable for kinetic parameter estimation in other ordered sequential processes, such as ERK dephosphorylation by dual specificity phosphatases (34, 35).

The fact that a well-studied model turned out to have an analytical solution was a pleasant surprise; nonetheless, the identification of key dimensionless groups is part and parcel of intelligent data analysis and could be attempted for a wider class of mechanisms. We found the two groups characterizing ordered dual phosphorylation through a nondimensionalization and steady-state approximation, which might be too cumbersome for larger systems. However, our model and kinetic data offer a clear test case for data-driven efforts to automate the discovery and estimation of effective parameters in biochemical networks. A recent study took a step in this direction, using low-dimensional description of parameter ensembles generated by local minimization algorithms (36).

Our phase representation of dual phosphorylation dynamics also proved useful in thinking about heterozygous systems, where two different enzyme variants are competing for access to the same substrate. This framework could prove particularly relevant for disorders like melanomas where one copy of MEK has become overactive through mutation. By using such a model to quantitatively determine how the dynamics of ERK activation differ in the presence of an activating MEK mutation, like MEK E203K, one could better predict the results of therapeutic interventions, like the introduction of a RAF (the kinase that phosphorylates MEK), MEK, or ERK inhibitor (37). When both enzyme variants follow the same mechanism, the heterozygous system is well-approximated by a homozygous system with appropriately chosen parameters. In other words, the presence of two enzyme variants does not introduce new effects. Future work will aim to find mechanisms where this is not true. In particular, we wonder whether one could provide an example where a system that can be ascertained to have only one stable steady state in homozygous conditions can give rise to multiple steady states or periodic oscillations when one of the reactions is catalyzed by two different variants. We leave this question as a challenge for scientists working on systems-level properties of biochemical networks (38, 39).

## Experimental procedures

All parameter values were determined using data from (19), in which 0.66 μ M MEK was combined with 5 μ M unphosphorylated ERK and concentrations of unphosphorylated (S_0_), monophosphorylated (S_1_), and dually phosphorylated (S2) ERK were tracked over 20 min, using Phos Tag Gels. Nonlinear least squares parameter fitting was done using the lsqcurvefit routine in MATLAB 2022a. The rate constants for the six-parameter model were found by fitting kinetic equations and the conservation equations for S_T_ and E_T_ to S_2_, S_1_, and S_0_ *versus* time, using the objective function $\sum_{i=1}^{21}\sum_{j=1}^{N}{\left(y_{ij}-x_{i}\right)}^{2}$, where $y_{ij}$ is the value of data point i (21 data points total with 3 phosphostates × 7 time points) for replicate j (N = 12 for WT MEK, N = 5 for MEK E203K, N = 5 for MEK Y130C, and N = 5 for MEK F53S) and $x_{i}$ is the value of data point i predicted by the model. For the two-parameter model, values for ϵ and δ were found by fitting the equation for the phase plane trajectory to S_1_ *versus* S_0_ data using the objective function $\sum_{i=1}^{7}\sum_{j=1}^{N}{\left(y_{ij}-x_{i}\right)}^{2}$, where $y_{ij}$ is the value of data point i (7 time points) for replicate j (N = 12 for WT MEK, N = 5 for MEK E203K, N = 5 for MEK Y130C), and $x_{i}$ is the value of data point i predicted by the model. For each model, 1000 fits were performed, with guesses selected by random sampling from a uniform or log-uniform distribution bounded by the values listed in Table S1.

## Data availability

All data are available in the manuscript.

## Supporting information

This article contains supporting information.

## Conflict of interest

The authors declare no conflicts of interest with the contents of this article.
