# Supplementary material for: Phase plane dynamics of ERK phosphorylation
Source: J Biol Chem. 2023 Sep 9;299(11):105234. doi: 10.1016/j.jbc.2023.105234 (PMC10616409; doi:10.1016/j.jbc.2023.105234)
Supplement: Supporting Figure S1 [file mmc1.docx]

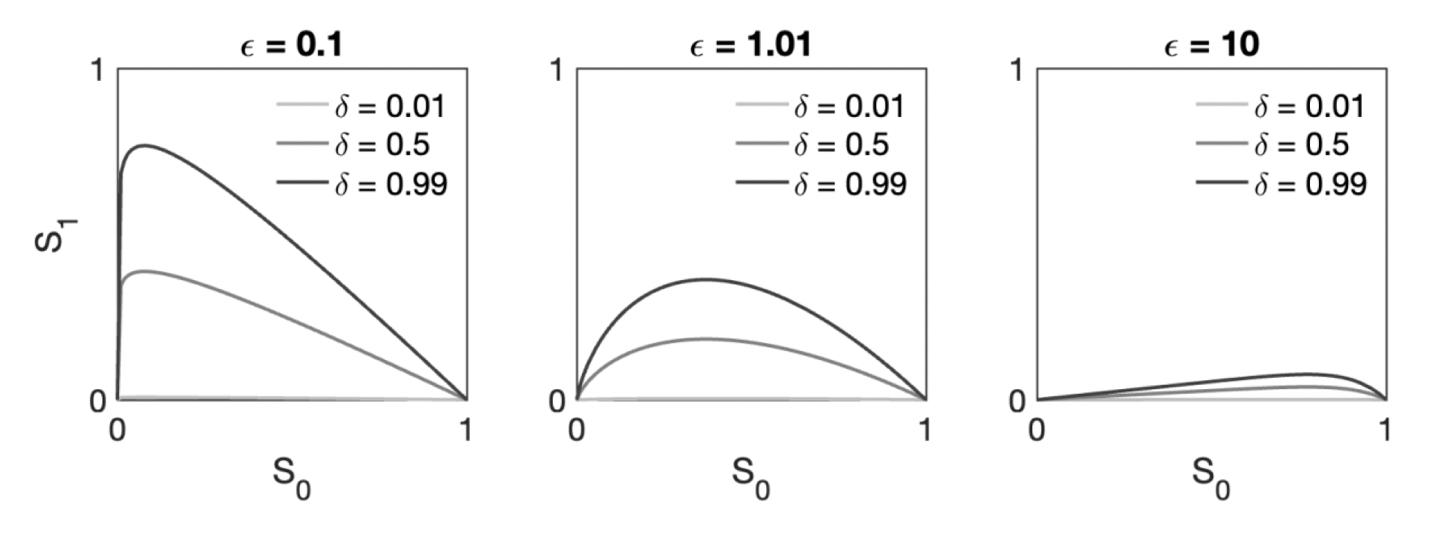
**Figure S1. The S1 vs. S0 phase plane trajectory shifts in response to changing values of** $\boldsymbol{\delta}$ **and** $\boldsymbol{\varepsilon}$**.** Values of $\varepsilon$ increase from left to right across plots, and values of $\delta$ increase from light gray to dark gray within each plot. In the case of distributive phosphorylation reactions, if $\varepsilon$ < 1, it indicates that the catalytic efficiency of the second phosphorylation is less efficient than that of the first phosphorylation. As shown in Fig. S1, more S_1_ is expected for a given amount of S_0_ as $\delta$ increases and the probability of complete phosphorylation occurring through distributive reactions increases. As $\varepsilon$ increases and the second phosphorylation becomes more catalytically efficient than the first, the ratio of S_1_ to S_0_ shifts such that less S_1_ is expected overall and more S_1_ is expected when S_0_ is high rather than when S_0_ is low, which is the case for low values of $\varepsilon$.
