# Supplementary material for: Phase plane dynamics of ERK phosphorylation
Source: J Biol Chem. 2023 Sep 9;299(11):105234. doi: 10.1016/j.jbc.2023.105234 (PMC10616409; doi:10.1016/j.jbc.2023.105234)
Supplement: Supporting Table S1 [file mmc2.docx]

**Table S1. Distributions and parameter bounds used to initialize parameter values for least-squares fitting.**

| **Parameter** | **Distribution** | **Lower Bound** | **Upper Bound** |
| --- | --- | --- | --- |
| $k_{f,1}$ | log uniform | 0.001 min^-1^ $\mu$M^-1^ | 1000 min^-1^ $\mu$M^-1^ |
| $k_{r,1}$ | log uniform | 0.01 min^-1^ $\mu$M^-1^ | 10000 min^-1^ $\mu$M^-1^ |
| $k_{cat,1}$ | log uniform | 0.01 min^-1^ $\mu$M^-1^ | 10000 min^-1^ $\mu$M^-1^ |
| $k_{f,2}$ | log uniform | 0.001 min^-1^ $\mu$M^-1^ | 1000 min^-1^ $\mu$M^-1^ |
| $k_{r,2}$ | log uniform | 0.01 min^-1^ $\mu$M^-1^ | 10000 min^-1^ $\mu$M^-1^ |
| $k_{cat,2}$ | log uniform | 0.01 min^-1^ $\mu$M^-1^ | 10000 min^-1^ $\mu$M^-1^ |
| $\epsilon$ | log uniform | 0.1 | 10 |
| $\delta$ | uniform | 0 | 1 |
