# Supplementary material for: Phase plane dynamics of ERK phosphorylation
Source: J Biol Chem. 2023 Sep 9;299(11):105234. doi: 10.1016/j.jbc.2023.105234 (PMC10616409; doi:10.1016/j.jbc.2023.105234)
Supplement: Supporting Figure S2 [file mmc3.docx]

**Figure S2. Relating the time-dependent and phase-plane representations of ERK phosphorylation kinetics.** A) Wild-type time-dependent and phase-plane trajectories. Time-dependent trajectories of S0 and S1 are shown on the two left-most panels, where the mean of the species concentration normalized by total substrate amount is plotted against time. The right-most panel depicts the same data plotted on the S1 vs. S0 phase plane. Error bars depict standard deviation (NWT=12, NE203K=5). The mean substrate concentration values are labeled t1-t7 (time points 1-7) to relate the data points between the time-dependent and phase-plane representation. In the phase-plane representation, measurements taken at earlier time points are always plotted to the right of measurements taken later, as S0 monotonically decreases over time. B) E203K time-dependent and phase-plane trajectories.
